# What Is Currently Known about the Role of CXCL10 in SARS-CoV-2 Infection?

**DOI:** 10.3390/ijms23073673

**Published:** 2022-03-27

**Authors:** Monika Gudowska-Sawczuk, Barbara Mroczko

**Affiliations:** 1 Department of Biochemical Diagnostics, Medical University of Bialystok, 15-269 Bialystok, Poland; mroczko@umb.edu.pl; 2 Department of Neurodegeneration Diagnostics, Medical University of Bialystok, 15-269 Bialystok, Poland

**Keywords:** SARS-CoV-2, COVID-19, chemokine, CXCL10, inflammation, cytokine

## Abstract

Dysregulation of the immune response plays an important role in the progression of SARS-CoV-2 infection. A “cytokine storm”, which is a phenomenon associated with uncontrolled production of large amounts of cytokines, very often affects patients with COVID-19. Elevated activity of chemotactic cytokines, called chemokines, can lead to serious consequences. CXCL10 has an ability to activate its receptor CXCR3, predominantly expressed on macrophages, T lymphocytes, dendritic cells, natural killer cells, and B cells. So, it has been suggested that the chemokine CXCL10, through CXCR3, is associated with inflammatory diseases and may be involved in the development of COVID-19. Therefore, in this review paper, we focus on the role of CXCL10 overactivity in the pathogenesis of COVID-19. We performed an extensive literature search for our investigation using the MEDLINE/PubMed database. Increased concentrations of CXCL10 were observed in COVID-19. Elevated levels of CXCL10 were reported to be associated with a severe course and disease progression. Published studies revealed that CXCL10 may be a very good predictive biomarker of patient outcome in COVID-19, and that markedly elevated CXCL10 levels are connected with ARDS and neurological complications. It has been observed that an effective treatment for SARS-CoV-2 leads to inhibition of “cytokine storm”, as well as reduction of CXCL10 concentrations. It seems that modulation of the CXCL10–CXCR3 axis may be an effective therapeutic target of COVID-19. This review describes the potential role of CXCL10 in the pathogenesis of COVID-19, as well as its potential immune–therapeutic significance. However, future studies should aim to confirm the prognostic, clinical, and therapeutic role of CXCL10 in SARS-CoV-2 infection.

## 1. Introduction

**Figure 1:**
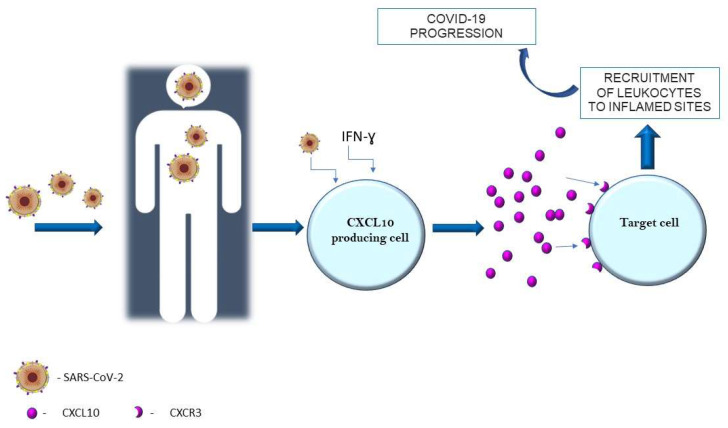
CXCL10 in SARS-CoV-2 infection.

In December 2019, a novel highly pathogenic coronavirus was discovered in patients with infectious respiratory disease in Wuhan, in the Chinese province of Hubei. The disease caused by Severe Acute Respiratory Syndrome Coronavirus-2 (SARS-CoV-2), nowadays known as the Coronavirus Disease 2019 (COVID-19), has spread rapidly around the world, triggering a pandemic [1]. It has been observed that the SARS-CoV-2 infection can cause a wide spectrum of clinical manifestations [2]. It is well known that COVID-19 in most cases is asymptomatic or mild with symptoms such as fever, dry cough, headache, muscle and joint pain, loss of smell and taste, and characteristic exhaustion and tiredness [3,4]. Unfortunately, sometimes SARS-CoV-2 infection may develop into a critical condition or even death in the course of an excess of the immune system. This can lead to serious tissue damage, as well as long-term complications of COVID-19 disease [5]. The SARS-CoV-2 coronavirus mainly affects the respiratory system of the patient via the angiotensin-converting enzyme 2 (ACE-2) receptors. In the respiratory system, by binding to the cells that build the alveoli of the lungs, it causes various symptoms, from completely non-threatening to respiratory failure requiring intensive care [6,7]. In the cardiovascular system, SARS-CoV-2 infection leads to complications such as infarction and inflammation of the heart muscle, exacerbation of heart failure, and arrhythmias [8]. On the other hand, the severe course of COVID-19 could be associated with serious neurological symptoms, including stroke, which indicates the entry of the virus into the central nervous system [9]. So, as can be seen, the SARS-CoV-2 virus can have an effect on every organ and the infection can lead to multisystem inflammatory syndrome. Although many different factors such as matrix metalloproteinases or vascular endothelial growth factors are involved in the course of immunological processes, undoubtedly one of the main regulatory factors are cytokines [10,11,12]. Cytokines are pleiotropic, which means that they can act on many different cell populations and exert different effects [13]. Long-term cytokine activity results in excessive levels of cytokines which are known as a “cytokine storm” [14]. An important group of cytokines are chemokines, which are classified according to their structure. A crucial role of chemokines in the recruitment of white blood cells to sites of inflammation [15]. For about two years, intensive research has been carried out on chemokines and chemokine receptors, which has resulted in the discovery of new functions and confirmation of their involvement in the development of COVID-19 disease. It has been observed that the expression of chemokines and their receptors can be positively or negatively regulated for their level of transcription by a variety of factors, including the SARS-CoV-2 virus [16,17,18,19]. Moreover, it has been identified from several studies that chemokine ligand 10 (CXCL10, IFN-γ-induced protein-10) is a very important factor that regulates many processes in the body, in particular, modulating the course and the intensity of inflammation caused by SARS-CoV-2 (Figure 1) [20,21,22]. In response to SARS-CoV-2 and increased activity of IFN-γ, this chemokine is produced by a wide range of cell types including neutrophils, monocytes, endothelial, or dendritic cells [23,24]. In turn, CXCL10 is selective ligand for CXCR3, which is mainly expressed on macrophages, T lymphocytes, dendritic cells, natural killer cells, and B cells. The CXCL10-CXCR3 axis has essential roles for the immune system. It has been revealed that normally it regulates immune cell differentiation, activation, and migration [25]. On the other hand, CXCL10 with CXCR3 have very important roles in selective cells recruitment to inflamed sites and in the intensification of inflammation, and also tissue damage. One of the main functions of CXCL10 is to promote the recruitment of CD8+ and Th1-type CD4+ effector T cells to infected or inflamed tissues. Moreover, it has been proven that CXCL10 promotes the accumulation of CD4+ and CD8+ T cells in infected tissues. Therefore, overactivity of the CXCL10–CXCR3 axis is associated with the development of some diseases. The abnormal activity of the axis has been observed in several autoimmune diseases including rheumatoid arthritis or systemic lupus erythematosus and cancers e.g., skin or brain cancers [26,27,28,29]. So, knowing that CXCL10 through CXCR3 is associated with inflammatory diseases, it has been suggested that the CXCL10–CXCR3 axis may be probably involved in development of COVID-19. Therefore, this review focused on the role of CXCL10 in SARS-CoV-2 infection.

## 2. Material and Methods

**Figure 2:**
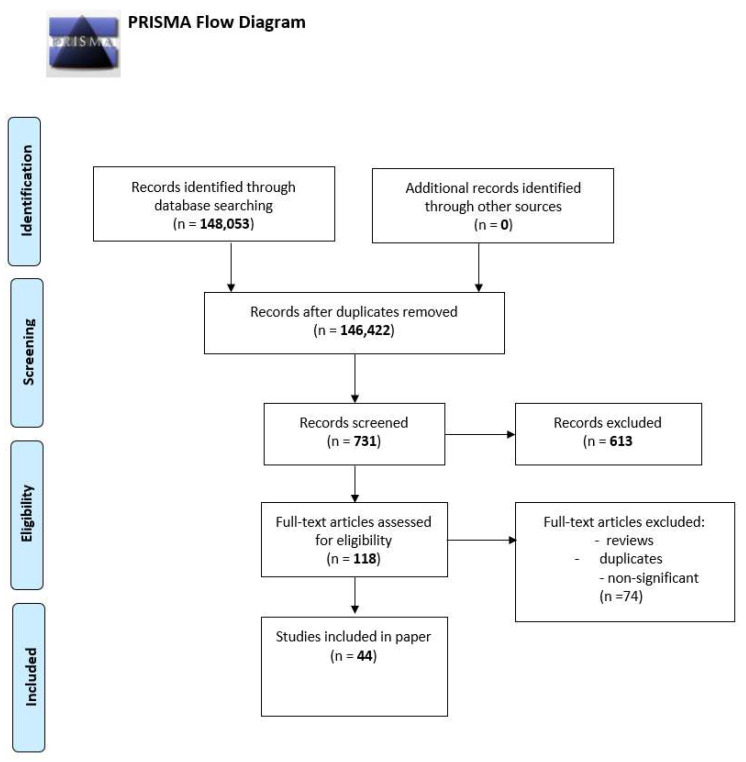
The PRISMA Flow Diagram.

We performed a comprehensive literature search covering the period up to the end of February 2022 using the MEDLINE/PubMed database with the following search strategy: key words “SARS-CoV-2” (148,053 studies). Then we used the key words “SARS-CoV-2 AND chemokines” and a total of 731 papers were found. A search including the key words “SARS-CoV-2 AND CXCL10” produced a total of 118 papers. In the next step, we limited studies to studies in English and we excluded duplicates or all non-significant papers (i.e., papers that did not concern CXCL10). Finally, 44 publications concerning CXCL10 were included in the review. All studies found were published between May 2020 and February 2022. All steps are presented in the PRISMA Flow Diagram (Figure 2) [30].

## 3. Results

### 3.1. Severity and Potential Risk Factors of the Disease 

Acute respiratory distress syndrome (ARDS) associated with COVID-19 can be considered a direct cause of death in many patients. It has been observed that ARDS caused by SARS-CoV-2 has different and distinctive pathogenesis in comparison to ARDS caused by e.g., bacterial infections. Therefore, Blot M. et al. compared cytokine response between COVID-19-related ARDS and non-COVID-19 ARDS. Using the Luminex assay, researchers measured the concentrations of 45 plasma and bronchoalveolar lavage fluid (BALF) biomarkers, including chemokines. They revealed that plasma and epithelial lining fluid CXCL10 concentrations were increased in group of patients with ARDS caused by COVID-19 in comparison to non-COVID-19 groups of patients. Moreover, CCL5, CXCL2, and CXCL1 concentrations were higher in patients with SARS-CoV-2 infection. Obviously, it was also observed that the number of ventilator-free days was higher in non-COVID-19 group in comparison to group of patients infected with SARS-CoV-2. It is worth pointing that only CXCL10 concentrations correlated with the number of ventilator-free days [31]. Going forward, it is well known that T lymphocytes are a very important component of the immunological system by, e.g., activation of other immune components and elimination of infected cells [32]. Therefore, taking into account that CXCL10 is the main ligand for CXCR3 receptor presented on effector T lymphocytes, it seems that the chemokine is probably one of the key mediators causing dysregulation of immune responses in COVID-19. It has been suggested that it may be a predictor of mechanical ventilation necessity in patients infected by SARS-CoV-2. Moreover, if a significant association between CXCL10 and COVID-19 immune response was observed, potential therapy targeting the CXCL10–CXCR3 axis should be take into consideration [31].

Ravindran et al. evaluated dynamics of immune response in patients with COVID-19. They measured the concentration of 48 proinflammatory factors in plasma samples from patients with SARS-CoV-2 infection. They observed that, at the time of hospital admission (one week after symptoms appeared), levels of cytokines and chemokines (CXCL10, eotaxin, G-CSF, Gro-α, CCL5, IL-2Rα, MCP-1, SCGF-β) were markedly elevated. Importantly, only CXCL10 levels fell after the patient’s condition improved. It was also observed that CXCL10 concentration was seven times higher in severe and almost four times higher in mild/moderate courses of COVID-19 in comparison to healthy controls. The level of CXCL10 differs also according to the severity of disease, being 100% higher in severe when compared to milder cases of COVID-19. The lower levels of chemokines after improvement of health and a mild course of disease indicate a reduction of inflammatory response [22]. So, it has been suggested that abnormal CXCL10 levels at hospital admission may predict COVID-19 outcome [21]. Similar results were obtained by other scientists who observed that patients with severe courses of COVID-19 have significantly elevated concentrations of CXCL10 [33,34]. 

Tripathy et al. compared pro-inflammatory cytokines and chemokines level between healthy, asymptomatic, and symptomatic patients. They revealed that besides cytokines such as IL-6 and TNF-α, CXCL10 was also increased in all above-mentioned patients with SARS-CoV-2 infection. Moreover, only CXCL10 was markedly elevated in symptomatic patients when compared to those without symptoms, and its concentration was higher in recovered patients in comparison to healthy ones. Summarizing, CXCL10 was the lowest in healthy patients, but higher in recovered and asymptomatic ones, and was the highest in patients with mild symptoms of SARS-CoV-2 infection. The differences were significant [35]. Also, another study revealed that patients with a severe course of COVID-19 have increased levels of CXCL10 [36]. The studies are evidence that show that production of CXCL10, with a high probability, increases the progression of COVID-19. However, in contrast to Ravindran et al., overexpression of CXCL10 about two months after the onset of symptoms of the disease was observed. This indicates that immunological reaction normalized for a longer time than observed improvement of patients’ conditions [35]. On the other hand, in the study by Kesmez Can et al., no significant differences of CXCL10 were observed among discharged and ex-patients in the severe group. However, as authors suggest, it may be related to the advanced age and small number of patients [36].

#### 3.1.1. Metabolic Aspects

Wang et al. tried to find the connection between CXCL10 and leptin, knowing that the obesity is associated with dysregulation of chemokines’ production and that leptin is linked to metabolic dysregulation and immune response within, e.g., T cells [33,37,38]. Interestingly, they revealed that there is association between chemokine and leptin in the context of the prediction of disease course or lymphocyte count [33]. Accordingly, it has been observed that patients with elevated body mass index (BMI) have died more often due to COVID-19 in comparison to those with normal BMI [39,40]. Földi et al. observed that obesity is a risk factor associated with admission to critical care units. Moreover, the requirement of mechanical ventilation is higher for patients with obesity [41]. In addition, in overweight non-survivors groups, the disease symptoms at admission were more intense. Basheer et al. also revealed the association between mortality in those patients’ group and cytokine storm syndrome. They showed that serum concentration of CXCL10 was almost three times higher at admission in comparison to samples taken after improvement of patients’ conditions. Moreover, CXCL10 levels were markedly elevated (4,5-fold) in the non-surviving group, which means in severe or critically ill patients, when compared to survivors. It seems that the results presented by Basheer et al. are in accordance to above described study by Maurya et al. [37,39]. 

#### 3.1.2. Gender

It has been observed that non-survivor patients are more often males [39]. It may be related to different immune response to SARS-CoV-2 between sexes. It is well known that monocytes play a role in the inflammatory and anti-inflammatory processes and monocytes are one of the major components of the innate immune system. Additionally, the CXCR3 receptor of CXCL10 is expressed by monocytes or macrophages and infections induce the synthesis of chemokines by human peripheral blood mononuclear cells (PBMCs). Knowing that SARS-CoV-2 drives e.g., monocytes to induce host immune response, it seems that COVID-19 progression is associated with overexpression of CXCL10. Therefore, Agarwal et al. tried to compare the immune response to SARS-CoV-2 of monocytes and dendritic cells between males and females. They isolated PBMCs from healthy patients and then the cells were infected by coronavirus. They observed that activation of monocytes in females in comparison to males was higher and PBMCs from females showed increased CD86 and HLA-DR on dendritic cells. In addition, the secretion of CXCL-10 by PBMCs was observed at 24 h, but the production of CXCL10 was significantly higher in males. Taking into account above, the results suggest that decreased activation of PBMCs and contrastingly increased synthesis of CXCL10 may be associated with the fact that males are non-survivors more frequently [42]. 

#### 3.1.3. Intestine

An interesting study conducted by Zhang et al. has demonstrated the effect of SARS-CoV-2 on gut microbiota composition and short chain fatty acid (SCFAs) metabolism. Scientists revealed that patients with COVID-19 had significant alterations in gut microbiota in comparison to the control group. Prolonged impairment of SCFAs and L-isoleucine biosynthesis has been observed in patients with COVID-19. In addition, lack of L-isoleucine and SCFA synthesis negatively correlated with the severity of disease. Surprisingly, there was also a negative correlation of L-isoleucine with CXCL10 and CRP plasma concentrations. Therefore, it has been speculated that gut microbiota plays an important role in dysregulation of the SARS-CoV-2-induced immune response. The conducted analysis and the obtained results indicate that gut microbiome may have a potential impact on host immunity and, inter alia, the level of CXCL10. Altogether, the imbalance between pro-inflammatory CXCL10 and impaired capacity for biosynthesis of anti-inflammatory SCFAs or L-isoleucine is probably an important factor affecting the COVID-19 severity [43].

#### 3.1.4. Nervous System

Speaking of post-COVID-19 complications usually mean respiratory complications. Meanwhile, it is already known that SARS-CoV-2 infection can lead to serious damage of the nervous system, both central (CNS) and peripheral (PNS). Probably, changes in nervous system are caused by an impaired inflammatory reaction. The entry of SARS-CoV-2 into the CNS is possible by ACE-2, neuropilin-1 (NRP-1), and transmembrane serine protease 2 (TMPRSS2) receptors that are expressed in olfactory epithelium of the nasal cavity and on the brain cells [44,45]. SARS-CoV-2, due to impairment of physiological cellular mechanisms and excessive activity of various cytokines and chemokines, attacks immune cells including macrophages, neutrophils, and T cells at the site of inflammation. Olivarria et al. performed research on mice, which suggested that CXCL10 plays an important role in neuroinflammation, consisting of mainly macrophages, monocytes, and T cells. It was observed that CNS infection of mice and viral replication in neurons were connected, with elevated expression of the following chemokines: CXCL10, CXCL9, CCL2, CCL5, and CCL19. However, the highest transcript levels were observed for CXCL10 and CXCL9. This can be explained by the fact that these particular chemokines are T lymphocyte chemoattractants, but interestingly, authors did not detect a robust T cell response in SARS-CoV-2 infection of CNS [46]. On the other hand, it has been revealed that increased levels of CXCL10 are associated with a Th17-mediated cytokine storm in SARS-CoV-2 [47,48,49]. 

Moreover, it has been observed that selected cytokines can cross the blood-brain barrier (BBB). Additionally, it has been revealed that CXCL10 can cause dysfunction of BBB and, as a result, increase the permeability of BBB, allowing coronavirus entry into the CNS. In summary, activated immune cells produce greater amounts of pro-inflammatory factors leading to cytokine storm [50]. Therefore, cytokine storm manifesting elevated levels of CXCL10 and coronavirus neuroinvasion are a possible reason for neurological manifestations and COVID-19-related severe nervous system complications.

#### 3.1.5. Association with Other Proinflammatory Factors

As mentioned above, CXCL10 is responsible for, inter alia, stimulation of monocytes or NK cells, migration of T lymphocytes, or modulation of the expression of adhesion molecules [51,52]. At the same time, it is known that CXCL10 or CRP-stimulated monocytes produce IL-6. Moreover, IL-6, similar to CXCL10, has the ability to affect T lymphocytes. For this reason, among others, some authors have tried to find a relationship between the concentration of CXCL10 and IL-6 in COVID-19 patients. It has been observed that both CXCL10 and IL-6 are increased and associated with systemic inflammation in SARS-CoV-2 infection [36,53,54]. Positive correlations between concentrations of CXCL10 and CRP, and between CXCL10 and IL-6 have been revealed. Moreover, the levels of CXCL10 and IL-6 are more than 10 times higher in patients with severe course and mild–moderate COVID-19 in comparison to healthy volunteers. Unfortunately, increased concentrations of CXCL10 and IL-6 were associated with poor prognosis [36]. 

Diagnostic significance of the most pro-inflammatory factors, CXCL10, TNF-α, IL-4, and IL-1β, has been evaluated. Tripathy et al. performed ROC curve analysis for all analytes, which generates the best cut-off points, potentially indicating that it could be used for differentiation of healthy and infected by SARS-CoV-2 asymptomatic and mildly symptomatic patients. At generated cut-offs for CXCL10 (124.9 pg/mL), TNF-α (29.19 pg/mL), IL-4 (1.395 pg/mL), and IL-1β (0.545 pg/mL), the area under the ROC curve (AUC) was the highest for CXCL10 (0.992) with the sensitivity of 97.3% and specificity of 91.7%. However, for all analyzed parameters, it was higher than 0.9. So, summarizing for CXCL10, it has the best diagnostic value, but all above-mentioned markers may be used for the diagnosis of SARS-CoV-2 infection [35].

### 3.2. Treatment

#### 3.2.1. Traditional Chinese Medicine

Unfortunately, there is no specific treatment available for COVID-19 and the therapy depends on the intensity of the symptoms. Nowadays, research is ongoing to develop new and the best COVID-19 therapies. Ma Q. et al. tried to evaluate the anti-inflammatory and antiviral effect of traditional Chinese medicine (TCM), including Liu Shen Capsule (LS) and Remdesivir. Researchers measured the effect of drugs on the African green monkey kidney epithelial cells (Vero E6) and human hepatocellular carcinoma cell lines (Huh-7) infected with SARS-CoV-2. They observed the reduction of SARS-CoV-2-induced cytopathic effect after 3 days incubation with TCM. Knowing that coronavirus induces strong inflammation reactions, they evaluated the expression of the most important pro-inflammatory cytokines, including CXCL-10 and CCL-2. Primarily, significantly elevated levels of chemokines in the virus group were reduced after treatment. Therefore, it seems that chemokines activity can be reduced by TCM. In addition, the level of CXCL-10 was the lowest after treatment with Remesdivir at5 µM, whereas CCL-2 was lowest after treatment with LS at a dose of2 µg/mL [55]. Scientists also studied the effect of Qingwenjiere (QJM) mixture on the human coronaviruses and, to our knowledge, the effect of QJM on SARS-CoV-2 has not been noted in other studies. Xie P. et al. used human rectal carcinoma cells (HRT-18), rhesus monkey kidney epithelial cells (LLC-MK2), Vero E6, and Huh-7 cells, and the cells were infected by four types of coronaviruses: HCoV-OC42, HCoV-229E, HCoV-NL63, and SARS-CoV, respectively. Then, the expression of various pro-inflammatory factors has been evaluated. In summary, QJM has been shown to be an effective drug that reduced virus-induced mRNA expression of, inter alia described in this review, CXCL10 [56]. Moreover, Lianhuaqingwen (LH) has been used to treat SARS-CoV-2 infection. This in vitro study shows the ability of LH to reduce replication of SARS-CoV-2 and production of CXCL10, as well as TNF-α and IL-6 at mRNA levels [57]. The changes of CXCL10 and other pro-inflammatory cytokines suggest that TCM may inhibit and block the cytokine storm caused by coronavirus infection. So, it seems that TCM have broad antiinflammatory and antiviral activity against human SARS-CoV-2. 

#### 3.2.2. Toll-like Receptors

Gene therapy is emerging as a treatment option in wide spectrum of diseases, and it was suggested that toll-like receptors’ (TLRs) gene modulation may be an effective option in COVID-19 treatment. Mammalian TLRs are proteins activated to help in the innate immune response, which is often the first line of the defense against infections and diseases [58]. The TLR family consists of 13 members, triggering the secretion of interferons and cytokines in response to viral infections, including SARS-CoV-2. It was observed that the toll-like receptor 7 (TLR7) gene is associated with poor prognosis among patients with COVID-19 [59,60]. In an interesting study conducted by Mantovani et al., it has been observed that stimulation of TLR7 in supernatants of stimulated PBMCs results in upregulation of 211 genes, including the CXCL10 gene, in comparison to unstimulated PBMCs. Moreover, authors identified loss-of-function variants of TLR7 in males with diagnosed COVID-19. The impaired signal pathways of TLR7 were associated with the changes of mRNA CXCL10. It was reported that the CXCL10 mRNA was significantly decreased in two rare variants of TLR7: 920Lys and Asp41Glu in patients with SARS-CoV-2 infection in comparison to healthy donors [61]. The Described study is in accordance with previous findings focusing on the role of TLR7 and CXCL10, which is directly associated with TLR7 in severe course of COVID-19 [59,61].

#### 3.2.3. Corticosteroids

It is well known that corticosteroids (CS) as antagonists for inflammation are used to treat severe COVID-19. It has been proven that corticosteroid therapy is the only treatment that significantly inhibits the inflammatory response and reduces mortality in COVID-19. Therefore, it has been recommended to treat critically ill patients with dexamethasone [62,63]. Martinez-Guerra et al. evaluated the effect of corticosteroid treatment on mortality of COVID-19 patients. They observed that in-hospital mortality was significantly lower in patients treated with dexamethasone in comparison to patients who did not receive corticosteroids. Moreover, the time to invasive mechanical ventilation initiation was longer in patients treated with CS [64]. This effectiveness of CS is probably associated with the inhibition of cytokine storm caused by SARS-CoV-2 infection. Knowing that SARS-CoV-2 can damage e.g., respiratory airways, including the trachea, by increased expression of CXCL10 hub gene, Zou et al. tried to evaluate the effect of CS on CXCL10. They observed that dexamethasone has a specific therapeutic effect on COVID-19 by regulating the CXCL10 and CXCR3 axis. The inhibition of the CXCL10/CXCR3 pathway reduces inflammation and cytokine storm in response to COVID-19. Summarizing, the overexpression of CXCL10 may be the main cause of chronic inflammation and tissue damage, but it can be effectively regulated with corticosteroids [65].

#### 3.2.4. Other

Knowing that actually used drug therapies are often ineffective and sometimes associated with unwanted side effects, researchers have tried to provide new information about the available potential therapeutic options against SARS-CoV-2. Therefore, the effectiveness of phosphodiesterase 4 (PDE4) inhibitors e.g., Tanimilast, has been evaluated. Nquyen et al. observed that PDE4 inhibitors effectively modulate SARS-CoV-2-induced inflammation and Th1-polarizing potential of dendritic cells. Going forward, it has been revealed that Th1-attracting CXCL10 or CXCL9 concentrations were reduced by Tanimilast. Knowing that CXCL10, on which this review focuses on, is one of the most important factors that amplify the immune response through the requirement of Th1 cells, thus, it was suggested that PDE4 inhibitors are a promising therapeutic option for COVID-19 [66]. Similar to the above-described drugs, *Pelargonium sidoides DC*. Root Extract EPs 7630 has an ability to reduce virus-induced immune response in human lung cells. The study showed that CXCL10 and other pro-inflammatory cytokines (e.g., CXCL9, IL-8, IL-13, TNF-α) were reduced as the result of EPs 7630 [67].

Different treatments affect patients’ bodies in different ways, but in case of COVID-19, the aim of the treatment is modulation of inflammatory response and reduction of viral load. Taking into account above-described results, CXCL10 modulation seems to be a promising effective therapeutic target in SARS-CoV-2 infection.

The summary of CXCL10 and other proinflammatory factors is presented in Table 1.

## 4. Conclusions

The impaired immune response plays an important role in the development and progression of SARS-CoV-2. It has been proven that CXCL10 is associated with inflammatory diseases, including COVID-19. As a result of inflammation, CXCL10 activates its receptor, CXCR3, which is mainly expressed on macrophages, T lymphocytes, dendritic cells, natural killer cells, and B cells. The upregulated activation of leukocytes leads to systemic inflammation, inducing, e.g., tissue damage. In this review paper, we focused on the changes in concentration and role of CXCL10 in COVID-19. Analyzed in this review, studies demonstrated that SARS-CoV-2 infection is associated with increased concentrations of CXCL10. CXCL10 levels differ also according to the severity of the disease and they is the highest in critically ill patients with COVID-19. Moreover, besides ARDS, inflammation of the nervous system is linked to increased synthesis of CXCL10. These findings indicated that a high level of CXCL10 as a result of SARS-CoV-2 infection should be considered as having a potentially high risk of complications or severe course of COVID-19. In addition, knowing that infection of SARS-CoV-2 induces “cytokine storm”, it has been suggested that the therapy of COVID-19 should focus on silencing the excessive inflammatory reaction. Modulation of CXCL10 activity seems to be a promising and effective therapeutic target of COVID-19. Therefore, more studies are needed to confirm the clinical, prognostic, and therapeutic role of CXCL10 in SARS-CoV-2 infection.

## Figures and Tables

**Table 1:** The significance of CXCL10 and other cytokines in SARS-CoV-2 infection.

CXCL10 Level	Other Cytokines	References
↑ in patients with ARDS	↑ CXCL1, CXCL2, CCL5	[24]
↑ in patients requiring mechanical ventilation		[24]
↑ at time of hospital admission	↑ Eotaxin, G-CSF, Gro-α, CCL5, IL-2Rα, MCP-, SCGF-b	[22,28,30]
↓ after improvement of patient’s condition		[22]
↑ in severe course od COVID-19		[26,27,33]
↑ asymptomatic and symptomatic patients	↑ IL-6, TNF-α	[32]
Higher in symptomatic patients in comparison to asymptomatic		[32]
Higher in asymptomatic and symptomatic patients in comparison to healthy controls	↑ IL-1β, IL-6, TNF-α	
↑ synthesis in males		[34]
↑ level is associated with systemic inflammation and poor prognosis	↑ IL-6	[33,37,38]
Negatively correlates with L-isoleucine		[39]
↑ in CNS infection	↑ CCL2, CCL5, CCL19, CXCL9	[42]
↓ after treatment with TCMs	↓ CCL2, TNF-α, IL-6	[47,48,49]
↓ after treatment with phosphodiesterase 4 inhibitors		[50]
Associated with TLR7 in severe course of COVID-19		[53,55]

## Data Availability

Not applicable.
